# Locally Aggressive and Multicentric Recurrent Extraocular Sebaceous Carcinoma: Case Report and Literature Review

**Published:** 2013-08-21

**Authors:** Advaith Bongu, Edward S. Lee, Stephen R. Peters, Ravi J. Chokshi

**Affiliations:** ^a^Department of Surgery, New Jersey Medical School, University of Medicine and Dentistry of New Jersey, Newark; ^b^Department of Pathology, New Jersey Medical School, University of Medicine and Dentistry of New Jersey, Newark

## Abstract

**Objective:** Sebaceous Carcinoma is a rare and aggressive malignant tumor. We present a case report of a large truncal tumor with multicentricity and aggressive locoregional recurrence that required trapezius myocutaneous flap reconstruction. Examining patterns of multicentricity, metastasis, and recurrence of sebaceous carcinoma in the literature we sought to explore potential reasons behind the aggressive behavior. **Methods:** Retrospective chart review was used to analyze the case in detail. Preoperative workup, intraoperative details, pathology, and follow-up visits were reviewed. Selected literature was considered with series of 5 or more patients. **Results:** The recurrent tumor was resected with negative margins leaving a defect of 14 × 7 cm^2^ that was covered with a trapezius myocutaneous flap. Postoperative hospital course was uneventful with no further local recurrence. On follow-up visits, tumors at other sites have been discovered. Reported rates of multicentricity, metastasis, and recurrence vary widely in the literature, but both subtypes of sebaceous carcinoma behave aggressively. **Conclusions:** A combination of genetic predisposition, delayed definitive care, and inherent tumor biology led to the aggressive locoregional disease in this case.

Sebaceous carcinoma is a rare but aggressive malignant tumor that can occur in extraocular and ocular variants. A genetic predisposition is present in the setting of the Muir-Torre syndrome. Other risk factors include previous radiation or oral thiazide.^1^ Most cases, however, are sporadic, presenting at ages between 60 to 80 years.^2^ Cases of sebaceous carcinoma have been reported since the 19th century but the work of Straatsma in the 1950s helped acknowledge sebaceous carcinoma as a separate and distinct entity.^3^

Sebaceous glands can be found on any hair-bearing region of the body and even within ectopic sites like the parotid.^1^ More than 70% of sebaceous carcinoma are found in the head and neck because of the high concentration of sebaceous glands. An important distinction is that the extraocular type includes these nonorbital head and neck tumors as well as those at all other sites throughout the body. Historically, the view has been that the ocular type was the more aggressive variant responsible for producing more widespread disease and metastases.^4^ Recent reports of highly aggressive extraocular tumors have called into question these views with reported rates of metastasis as high as 21%.^5^ We present a case report of an extraocular variant and examine the unique features of multicentricity and aggressive locoregional recurrence in the absence of widely metastatic disease.

## CASE REPORT

A 47-year-old Hispanic man initially presented in Mexico 6 years prior to referral to our office. In Mexico, he had sebaceous cyst like masses, which were removed from his upper back and left chest. The patient was unaware of the pathologic diagnosis and healed uneventfully. Years later, appearance of larger lesions of a similar character in the same anatomic locations prompted evaluation at a community hospital in the United States. When an excisional biopsy of both sites revealed sebaceous carcinoma with positive margins, he was referred to our institution for evaluation and management.

**Figure 1:**
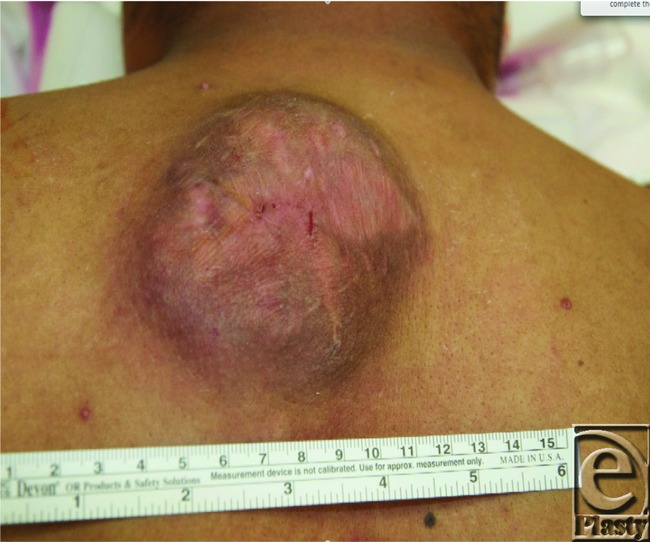
Tumor appearance.

On presentation, his primary complaint was discomfort and the slow steady growth in size of his upper back lesion (Fig 1). No other significant medical history and family history were reported. On examination, he had a well-healed anterior chest wall incision and an approximately 9 × 9 cm^2^ tender, raised heterogeneous region on his upper mid back. He underwent a fludeoxyglucose-positron emission tomographic (FDG-PET) scan and an endoscopic workup to rule out Muir-Torre syndrome. The FDG-PET scan indicated only avidity in the back mass, and the endoscopic workup was negative for any additional findings.

**Figure 2:**
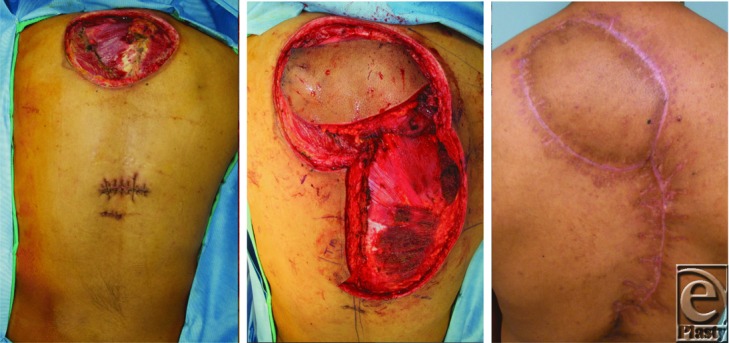
Radical resection, myocutaneous reconstruction, and healed incision.

The patient was then scheduled for a wide local excision of his back and chest lesions with planned defect reconstruction by plastic surgery. First, the preexisting scar on the chest was excised with 2 cm margin and primarily closed. The back lesion required a radical resection due to invasion into the trapezius muscle. A 14 × 7 cm^2^ area was ultimately resected and a temporary vacuum dressing was placed over the large back defect awaiting final pathologic margins. A smaller 0.5-cm lesion identified on his inferior back was also removed and closed primarily. One week later, he underwent a trapezius myocutaneous flap with local tissue rearrangement for coverage (Fig 2). On subsequent postoperative visits in the weeks following, more small nodular lesions were identified on his back with varied pathologies from basal cell carcinoma to a new sebaceous carcinoma nodule. He was later found to have Muir-Torre syndrome–related genetic mutations.

## DISCUSSION

Initially extraocular tumors were thought to be more indolent, but the paradigm has shifted with more reported cases toward recognizing that both extraocular and ocular subtypes can behave aggressively. The incidence of sebaceous carcinoma has increased overall but the disease itself is still rare. Looking at Surveillance, Epidemiology and End Results cancer registries from the years 1973 to 2003, the incidence of all combined sebaceous carcinoma grew by 5.2% per year,^6^ but there were still fewer than 2000 cases identified in the United States over a period of 34 years.^7^ Muir-Torre syndrome is even rarer with only a few hundred cases reported worldwide.^8^ Much of the literature is in the form of a few hundred case reports^9^ with limited pooled and larger series. Registry data has shown that the 5-year overall survivals ultimately do not differ for the ocular and nonocular subtypes (75.2 vs 68.0 years, *P* = .66, n = 1349).^10^ Behavior with respect to recurrence, multicentricity, and metastasis have been harder to generalize from the literature (Table 1). Incidences were included in Table 1 when reported or calculable. Recurrence refers to local disease after an attempted intervention. Multicentricity was defined as occurring simultaneously at noncontiguous sites which in ocular tumors constitutes involvement of the upper and lower eyelids. Metastasis includes both distant and regional disease. The widely differing rates depicted have to be viewed considering underlying differences in study design, patient selections compounded by pooling data, tumor size/grade, and surgical techniques. Nevertheless, it again brings to light the aggressive potential of both subtypes.

The aggressive nature of this case is likely multifactorial in origin with contributions from a genetic predisposition, delayed definitive treatment, and tumor biology. Muir-Torre syndrome is an autosomal dominant disease defined by the presence of a single sebaceous gland neoplasm and a minimum of one internal malignancy, usually gastrointestinal or genitourinary. A variant of the hereditary nonpolyposis colorectal cancer syndrome, Muir-Torre syndrome has similar mutations in mismatch repair genes and microsatellite instability. In a series of 120 patients, the cutaneous lesions occurred prior to diagnosis of an internal malignancy in only 41%.^22^ Muir-Torre syndrome visceral cancers therefore can occur in a synchronous or metachronous fashion. Although multiple genetic targets can be affected specifically, the MSH2 mismatch mutation on immunohistochemical analyses of extraocular tumors is highly suggestive of Muir-Torre syndrome.^23^ It is thought that the pathogenesis may involve multipluripotent stem cells in the skin that over time accumulate mutations necessary for tumorigenesis. These could also theoretically play a role in recurrence.^24^ Those with Muir-Torre syndrome have been described as having “cancers in a can.” Muir-Torre syndrome patients may have cancers years later due to this shared microsatellite instability in all tumors. Patients with sebaceous carcinoma have a 43% increased risk of developing a new malignancy compared with the general population.^6^

An issue in this case was the patient's delay in diagnosis and definitive treatment. Because of variable clinical presentations, sebaceous carcinoma can be difficult to diagnose on physical examination. The orbital tumors, for example, may be clinically indistinguishable from more benign pathology or inflammatory conditions. Nelson^3^ estimated that this added at least 1 to 2.9 years to the diagnosis. The clinical appearance of the extraocular tumors is also not pathognomonic, usually presenting as a reddish yellow nodule of varying diameter.

Unfortunately, the histologic diagnosis of both types is no more straightforward. While the poor prognostic histologic features of sebaceous carcinoma are universally agreed upon, these tumors under a microscope may resemble other cutaneous malignancies.^3^ In a series of 60 cases of sebaceous carcinoma of the eyelid, the diagnosis was only suspected one third of the time and even after biopsy review was made histopathologically only in 50% of cases. In fact, 18% were even misdiagnosed as squamous cell carcinoma.^14^ Kazakov^21^ published a case series of a unique previously unrecognized pathological presentation of sebaceous carcinoma in a pseudorosette carcinoid fashion. In his case, the sebaceous differentiation only became apparent in a recurrent lesion. Whether or not the diagnosis was made available after the initial resection in Mexico or if socioeconomic factors played a role is unknown.

**Figure 3:**
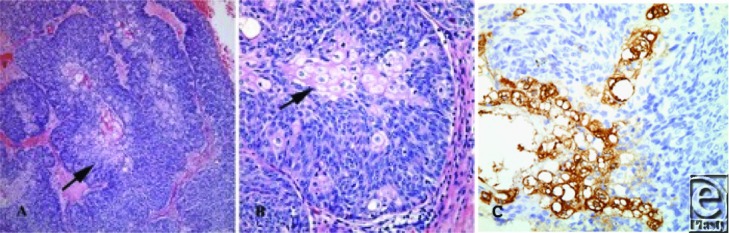
(*a*) 100× magnification showing the basaloid proliferation of neoplastic cells in broad lobules with pale central areas of sebaceous differentiation (arrow). (*b*) 400× magnification highlighting areas of abrupt sebaceous differentiation (arrow). (*c*). An epithelial membrane antigen immunoperoxidase stain with characteristic brown staining of cells with sebaceous differentiation.

Histopathological findings can influence tumor behavior. When eyelid tumors were found to be more likely to have distant metastases at presentation, this subset was more often poorly differentiated (49.8%) when compared to other head and neck sites (22.7%) and all other sites (16.7%).^7^ The poor prognostic indicators histologically are multicentricity, poor sebaceous differentiation, pagetoid distortion of overlying epithelium, and an infiltrative growth pattern with invasion of vasculature or lymphatics.^16^ Pagetoid distribution is unique to sebaceous carcinoma. Ocular adnexa cases with pagetoid invasion were found to have a mortality of approximately 50%, while those without invasion were associated with an 11% mortality.^16^ Deo,^20^ looking at 13 locally advanced ocular sebaceous carcinomas requiring orbital exenterations, found that his rate of recurrence with negative margins was 54%. Although this rate is higher than expected, it reflects the fact that a highly infiltrative growth pattern will recur.^20^ On microscopic examination, both specimens in this case demonstrated basaloid proliferations of neoplastic cells with abrupt differentiation into cells with copious amounts of vacuolated cytoplasm characteristic of sebaceous differentiation (Fig 3).

A final explanation for more aggressive tumors lies with the interventions themselves. When disease has been present for a long period or there is repeat treatment/trauma to the site, this can increase the potential to develop skip lesions and multicentricity.^17^ Multicentricity provides the most plausible route for recurrence, but it is often difficult to know whether it was not a de novo lesion instead. True multifocal dermal sebaceous carcinoma is rare, reported to range from 2% to 12%, and only occurs in advanced presentations.^17^ Consider also that the basis for the literature defined margins when excising these tumors was a retrospective study of 14 patients by Dogru which demonstrated recurrence with a surgical margin of 1 to 3 mm, but no recurrence if 5 mm or greater.^18^ Ignoring for a moment that it is possible that margins were left positive at the time of the initial excision, local recurrence is even associated with wide surgical excision and Mohs Micrographic Surgery with acceptable margins. 9% to 36% of sebaceous carcinoma patients have local recurrence within 5 years after wide local excision and as early as 3 to 35 months after Mohs.^3^

## CONCLUSION

A combination of genetic predisposition, delayed definitive care, and inherent tumor biology led to the aggressive locoregional disease in this case. While the diagnosis of Muir-Torre syndrome involves the association of sebaceous carcinoma and one visceral neoplasm, a negative FDG-PET scan and endoscopy do not entirely rule this out. Our patient has evidence of genetic markers for Muir-Torre syndrome and may develop a visceral neoplasm in the future. In addition, the likelihood increases when sebaceous carcinoma is diagnosed before the age of 50. Vigilance and close surveillance will be of utmost importance for this patient.

## Figures and Tables

**Table 1:** Selected series reporting recurrence, multicentricity and metastasis

Author	N	Pooled?	Time period	Type*	Recurrence	Multicentricity	Metastasis
Wick et al^11^	5	No	1905-1983	EO	20% (1/5)	40% (2/5)	60% (3/5)
Rulon and Helwig^12^	59	Yes	1942-1973	EO	19%(11/59)	…	14% (8/59)
Bailet et al^5^	91	Yes	1955-1990	EO	29%(26/91)	…	21%(23/83)
Dowd et al^2^	25	No	1973-2007	EO/O	4% (1/25)	…	12% (3/25)
Dasgupta et al^10^	1349	Registry	1973-2004	EO/O	…	29% (404/1349)	1.7% (23/1349)
Pardo et al^13^	30	No	1974-1986	O	…	17% (5/30)	20% (6/30)
Shields et al^14^	60	No	1974-2003	O	18% (11/60)	…	8% (5/60)
Zurcher et al^15^	43	No	1976-1992	O	14% (6/43)	7% (3/43)	14% (6/43)
Rao et al^16^	104	No	1982	O	22% (23/104)	17% (18/104)	22% (23/104)
Snow et al^17^	49	Yes	1985-2001	O	38% (10/26)	6% (3/49)	8% (4/49)
Dogru et al^18^	14	No	1986-1994	O	36% (5/13)	…	…
Bassetto et al^19^	5	No	1994-2004	EO	20% (1/5)	…	40% (2/5)
Deo et al^20^	13	No	1997-2010	O	54% (7/13)	15% (2/13)	77%(10/13)
Kazakov et al^21^	7	No	2005	EO	17% (1/6)	…	0% (0/6)

* EO indicates extraocular; O, ocular subtypes.
